# Anti-Inflammatory Effects of IL-27 in Zymosan-Induced Peritonitis: Inhibition of Neutrophil Recruitment Partially Explained by Impaired Mobilization from Bone Marrow and Reduced Chemokine Levels

**DOI:** 10.1371/journal.pone.0137651

**Published:** 2015-09-11

**Authors:** Ralf Watzlawick, Elisabeth E. Kenngott, Francesca Diane M. Liu, Jan M. Schwab, Alf Hamann

**Affiliations:** 1 Department of Neurology and Experimental Neurology, Charité Campus Mitte, Clinical and Experimental Spinal Cord Injury Research Laboratory (Neuroparaplegiology), Charité–Universitätsmedizin Berlin, Berlin, Germany; 2 Department of Experimental Rheumatology, Deutsches Rheuma-Forschungszentrum, Berlin, Germany; 3 Department of Rheumatology and Clinical Immunology, Charité–Universitätsmedizin Berlin, Berlin, Germany; 4 Department of Neurology, Spinal Cord Injury Division, The Neuroscience Institute, The Ohio State University, Wexner Medical Center, Columbus, Ohio, United States of America; 5 Department of Neuroscience and Center for Brain and Spinal Cord Repair, Department of Physical Medicine and Rehabilitation, The Neuroscience Institute, The Ohio State University, Wexner Medical Center, Columbus, Ohio, United States of America; University of Leuven, Rega Institute, BELGIUM

## Abstract

Rapid activation of the innate immune system is critical for an efficient host response to invading pathogens. However, the inflammatory reaction has to be strictly controlled to minimize harmful immunopathology. A number of mediators including the cytokine interleukin-27 (IL-27) appear to be responsible for limitation and resolution of inflammation. Despite increasing knowledge of its suppressive effects on T cells, the influence on neutrophils and macrophages is poorly understood. To determine the role of IL-27 in innate immune responses we analysed the effect of IL-27 in a T cell independent model of zymosan-induced peritonitis. Early administration of recombinant IL-27 strongly reduced the number of neutrophils recruited to the peritoneal cavity after zymosan application as well as the neutrophil frequency in the blood. Simultaneously, IL-27 reduced the release of neutrophils from the bone marrow upon inflammation. Although cytokine levels were not affected by IL-27 treatment, the levels of the chemokines KC, MCP-1 and MIP-1α in the peritoneal fluid were strongly decreased. These findings demonstrate that IL-27 is able to control mobilisation and recruitment of neutrophils into the peritoneal cavity and identify a novel mechanism to limit inflammation caused by innate immune cells.

## Introduction

IL-27, a multifaceted cytokine, principally exerts both stimulatory and inhibitory actions within the immune system. Most *in vivo* studies, however, have highlighted its role in dampening immune responses and limiting inflammation. Indeed, IL-27 is a key inducer of the inhibitory cytokine IL-10 in T cells which in turn suppresses the pro-inflammatory cytokine IL-17. Moreover, IL-27 is able to directly suppress the production of IFNγ and other inflammatory cytokines in T cells [1–3]. While the effects of IL-27 on T cells are well studied, increasing evidence suggest additional, poorly investigated regulatory effects on other cells, notably cells of the innate system.

IL-27 is a member of the IL-12 superfamily and consists of two subunits, the EBI-3 and p28 chain [4]. The heterodimeric IL-27 interacts with the IL-27R complex composed of IL-27Ra (also known as WSX-1 or TCCR) and gp130, being expressed on numerous cell types of the immune system including T and B cells, monocytes, macrophages, dendritic cells (DCs), mast cells as well as on neutrophils [5, 6]. IL-27 is predominantly produced by macrophages and DC as result of type I interferons, prostaglandins, or pathogen-associated signals including Toll-like-receptor 2 (TLR2) triggering [7–9] and was shown to be produced with a delayed kinetic during acute inflammation compared to inflammatory cytokines [10].

Consistent with its anti-inflammatory function, treatment with IL-27 was able to ameliorate disease in the experimental autoimmune encephalomyelitis (EAE) model by suppressing IL-17 [11]. In influenza, application of IL-27 in a late phase of infection improved survival by reducing immunopathology and monocyte / neutrophil infiltration into the lung [10]. Interestingly, the protective effect in this model was not due to modulation of the T cell response, but rather appeared to involve actions on innate cells. Indeed, a few studies report effects of IL-27 on macrophages [12], neutrophils [13] or DCs [14].

To further elucidate the anti-inflammatory effects of IL-27 on the innate immune system *in vivo*, we chose the zymosan-induced peritonitis as a robust model of acute innate inflammation. In this model, injection of the TLR2 ligand zymosan into the peritoneal cavity leads to an inflammatory reaction and immediate recruitment of innate immune cells to the site of inflammation. The self-resolving response mimics all the features of acute inflammation including pain, leukocyte infiltration and synthesis of inflammatory mediators [15].

The hallmark of an acute inflammation is the accumulation of high numbers of neutrophils at the site of infection [16]. Neutrophils are among the first line of defense upon acute infection and are recruited rapidly to the site of inflammation [17]. The bone marrow is the largest reservoir of neutrophils; it is estimated that, under homeostatic conditions, approximately 90% of neutrophils reside in the bone marrow [18]. Inflammatory mediators initiate increased release from the bone marrow and an initial activation of neutrophils, which allows them to infiltrate inflamed tissue and extend their usually short lifespan [19]. Although neutrophils play a crucial role in fighting acute infections and clearing pathogens, these cells also contribute to tissue immunopathology due to their histotoxic capacity. Neutrophil homeostasis is therefore tightly regulated within healthy individuals showing a high turnover rate of neutrophils in the circulation. Mediators controlling the function of neutrophils are, however, so far poorly defined.

Here, we demonstrate that IL-27 treatment inhibits neutrophil accumulation at the site of inflammation in the T cell independent model of zymosan-induced peritonitis. Our findings suggest that IL-27 can act as major regulator of the migration of neutrophils from the bone marrow to the site of inflammation by reducing chemokine production and by suppressing their mobilization from bone marrow.

## Materials and Methods

### Ethics statement

All procedures were performed in accordance with the German animal welfare legislation and the guidelines from the European Union and the European convention for the protection of vertebrate animals used for experimental and other scientific purposes. Experimental procedures were approved by the ethics committee and the Berlin state authorities (LAGeSo #G203/11).

### Mice

Male C57BL/6 mice, 6–10 weeks old, weighing 20–25 g, were purchased from Charles River, Sulzfeld, Germany. Mice were randomly assessed to the different experimental groups and maintained under specific pathogen-free conditions.

### Peritonitis induction and application of IL-27

Zymosan A (Sigma Z-4250, Saccharomyces cerevisiae) powder was suspended in PBS (1 mg/ml), sonicated and 1ml was injected intraperitoneally (i.p.) after the mice were anesthetized by isoflurane (1.5–2% in 2:3 oxygen/nitric oxide, inhaled). 200 ng recombinant IL-27 (R&D Systems) was injected i.p. 12 h prior to the administration of zymosan for the preIL-27 group and simultaneously for the IL-27 group. The control group (zym) received the equivalent volume of PBS instead of IL-27. Vehicle-treated animals (vehicle) received equivalent volumes of PBS instead of zymosan or IL-27 and were sacrificed 12h post-zymosan.

### Tissue preparation

Mice were sacrificed by inhalation of isoflurane 4, 12, 24 or 48 hours after the zymosan injection, respectively. 5 ml peritoneal lavage, blood and tibial and femoral bone marrow were obtained and stored on ice. Samples were blind-sided to avoid biased analysis. Aliquots of the lavage fluid, blood and bone marrow were stained with Turk’s solution and differential cell counts were performed twice using a Fuchs Rosenthal hemocytometer and a light microscope. Total number of cells in lavage and blood were calculated for the collected lavage volume (5 ml) and 1 ml of blood.

### Antibodies and flow cytometry analysis

For flow cytometric analysis of cell-surface markers, cells were stained with murine monoclonal antibodies in PBS containing 0.1% BSA and 20 μg/ml anti Fcγ-receptor. The following murine monoclonal antibodies were used: from eBioscience, CD11b (M1/70), CD11c (N418), NK1.1 (PK136); from from BD Biosciences and Biolegend, GR1 (RB6-8C5) and CD19 (6D5), respectively. CD3 (145-2C11), B220 (RA3.6B2) and anti-Macrophage marker (F4/80) were produced in-house (Deutsches Rheuma-Forschungszentrum). Prior to flow cytometric analysis, erythrocytes in blood samples were lysed using ADG-Lyse solution (Nordic-Mubio, Susteren, Netherlands). Flow cytometry data were acquired on a FACSCanto II (Becton Dickinson) and analysed with FlowJo Software (Version 9.7.5 for MacOS, TreeStar).

### Cytokine and chemokine detection

Cytokine concentration in peritoneal lavage was measured by ProcartaPlex multiplex immunoassay (eBioscience) according to manufacturer´s instructions using a Bio-Plex 200 System (BioRad). Chemokine concentration in peritoneal lavage, blood and bone marrow was assessed by FlowCytomix multiple detection kit (eBioscience) according to manufacturer`s instructions and analysed by flow cytometry using a FACSCanto II (Becton Dickinson).

### FACS and Statistical Analyses

FACS plots for bone marrow samples are displayed as frequency of the analysed cells; results for blood cells are displayed as cell number per ml blood and results for peritoneal lavage are displayed as total cell number in obtained lavage. Statistical analyses were performed with Prism 6 for Windows (Graphpad Software Inc.): Data are means ± S.E.M. unless stated otherwise. Statistical tests used include D’Agostino and Pearson omnibus-normality test and Mann-Whitney t test. P-values of less than 0.05 were considered statistically significant.

## Results

### Treatment with IL-27 decreases neutrophil numbers in the peritoneal cavity after induction of peritonitis

**Fig 1:**
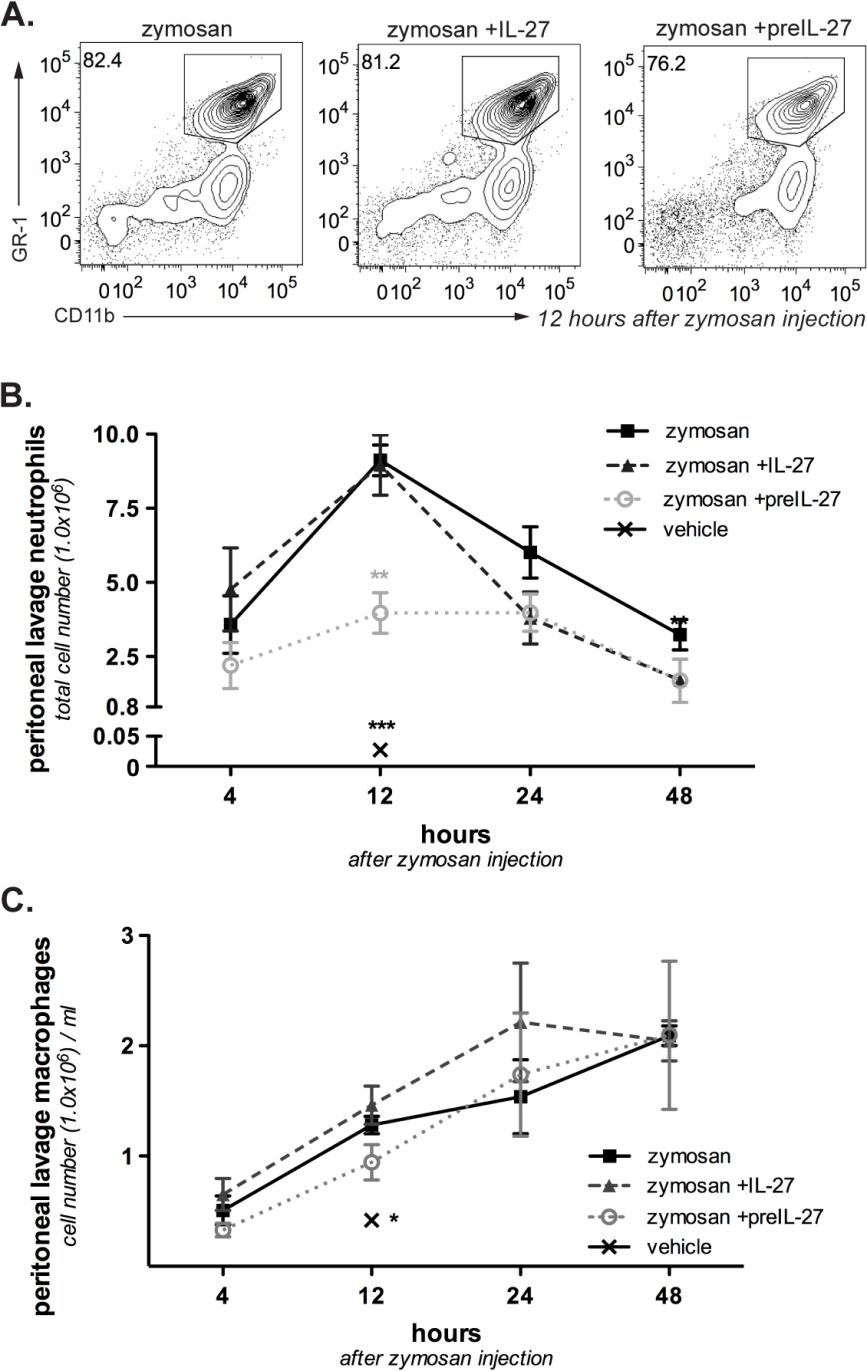
Influence of IL-27 on the inflammatory response in sterile zymosan-induced peritonitis. IL-27 was either given together with zymosan (zymosan +IL-27) or 12 h before zymosan application into the peritoneum (zymosan +preIL-27). The control group received zymosan only. **A.** Representative FACS plots for peritoneal neutrophils and macrophages isolated from the peritoneal lavage 12h after induction of peritonitis. GR1+CD11b+ cells are defined as neutrophils, CD11b+ single positive cells as macrophages. Cell frequencies within the neutrophil gate are depicted. **B.** Absolute number of neutrophils in the peritoneal lavage at various time points after zymosan injection. Results are pooled from four independent experiments and represent mean ± S.E.M (n = 4–12). **C.** Absolute number of macrophages in the peritoneal lavage at various time points after zymosan injection. Pooled data from four independent experiments, mean ± S.E.M. (n = 4–12). Mann–Whitney test was used to compare between groups, *p<0.05, ** p<0.01.

To evaluate the anti-inflammatory potential of IL-27 we administered IL-27 i.p. simultaneously to the zymosan injection (zymosan + IL-27). One control group received zymosan and PBS instead of IL-27 treatment, another group received PBS as a vehicle only to monitor homeostatic cellularity. While only minimal numbers of neutrophils were detected in the peritoneum of the vehicle-only treated group (vehicle 2.7x10^4^ ± 1.5x10^4^), injection of zymosan led to an major recruitment of GR1^+^/CD11b^+^ neutrophils in the peritoneal cavity that peaks at 12 h post-zymosan injection (Fig 1A and 1B), in line with the classical kinetics described elsewhere [20]. Although IL-27 and zymosan co-injection did not affect neutrophil recruitment within the 12 h initial response, neutrophil numbers are reduced at later time points. Simultaneous IL-27 treatment therefore seems to affect the innate immune response only several hours after application. To determine whether early administration of IL-27 can suppress zymosan-induced neutrophil recruitment to the peritoneum, we administered IL-27 12 h prior to zymosan injection. In contrast to the simultaneous treatment, we observed significantly decreased neutrophil numbers in the peritoneum for the pre-injected group (zym 9.1x10^6^ ± 0.5x10^6^, zym+preIL-27 4.0x10^6^±0.6x10^6^; mean ± SEM). Thus, IL-27 treatment is able to reduce neutrophil accumulation in the peritonitis model, but effects show a lag phase of several hours.

After the peak at 12 h, neutrophil numbers declined faster in the groups treated with IL-27, resulting in significantly lower cell numbers in both IL-27-treated groups at 48 h (zym, 3.2x10^6^ ± 0.5x10^6^, zym+preIL-27, 1.7x10^6^±0.7x10^6^, zym +IL-27, 1.7x10^6^±0.2x10^6^). This could suggest that treatment with IL-27 not only reduced recruitment of neutrophils to the peritoneum but also affected the resolution phase after the peak of inflammation.

### Treatment with IL-27 does not influence other cell populations within the peritoneal cavity

**Fig 2:**
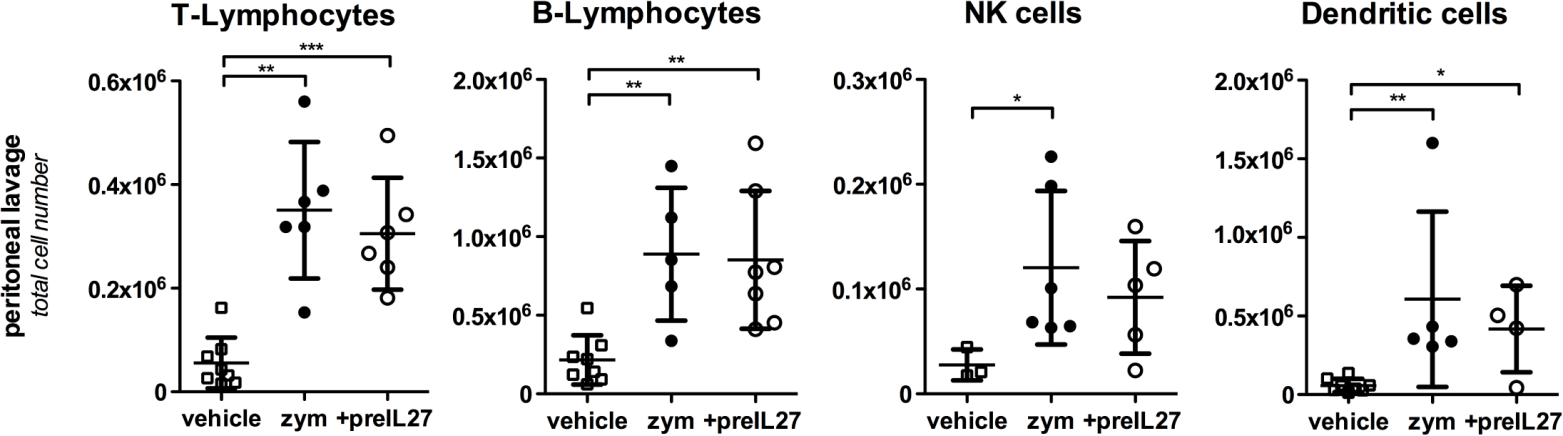
IL-27 pre-treatment does not affect the cell numbers of T, B, NK and dendritic cells in the peritoneal lavage. Cells in the peritoneal lavage were collected 12 h after the administration of zymosan with or without pre-treatment with rIL-27 and measured by FACS analysis. Results from three different experiments are combined, given are means ± S.D (n = 4–9). Mann–Whitney test was used to compare between groups, *p<0.05, ** p<0.01, ***p<0.005.

Accumulation of CD11b^+^/GR1^-^ macrophages in the peritoneal lavage steadily increased post-zymosan application (Fig 1C), consistent with the different kinetics of neutrophils and monocytes [15]. In contrast to neutrophils, neither IL-27 pre-treatment nor co-injection suppressed macrophage accumulation in the peritoneum (at 12 h: zym 1.3±0.08, zym+IL-27 1.5±0.2, zym+preIL-27 0.9±0.2, vehicle 0.4±0.1, cell numbers x10^6^). Similarly, numbers of lymphocytes, natural killer cells (NK) and DCs being recruited into the peritoneum were not significantly affected by IL-27 treatment, but considerably increased compared to vehicle-injected animals (Fig 2).

### IL-27 treatment decreases neutrophil levels in the blood and bone marrow

**Fig 3:**
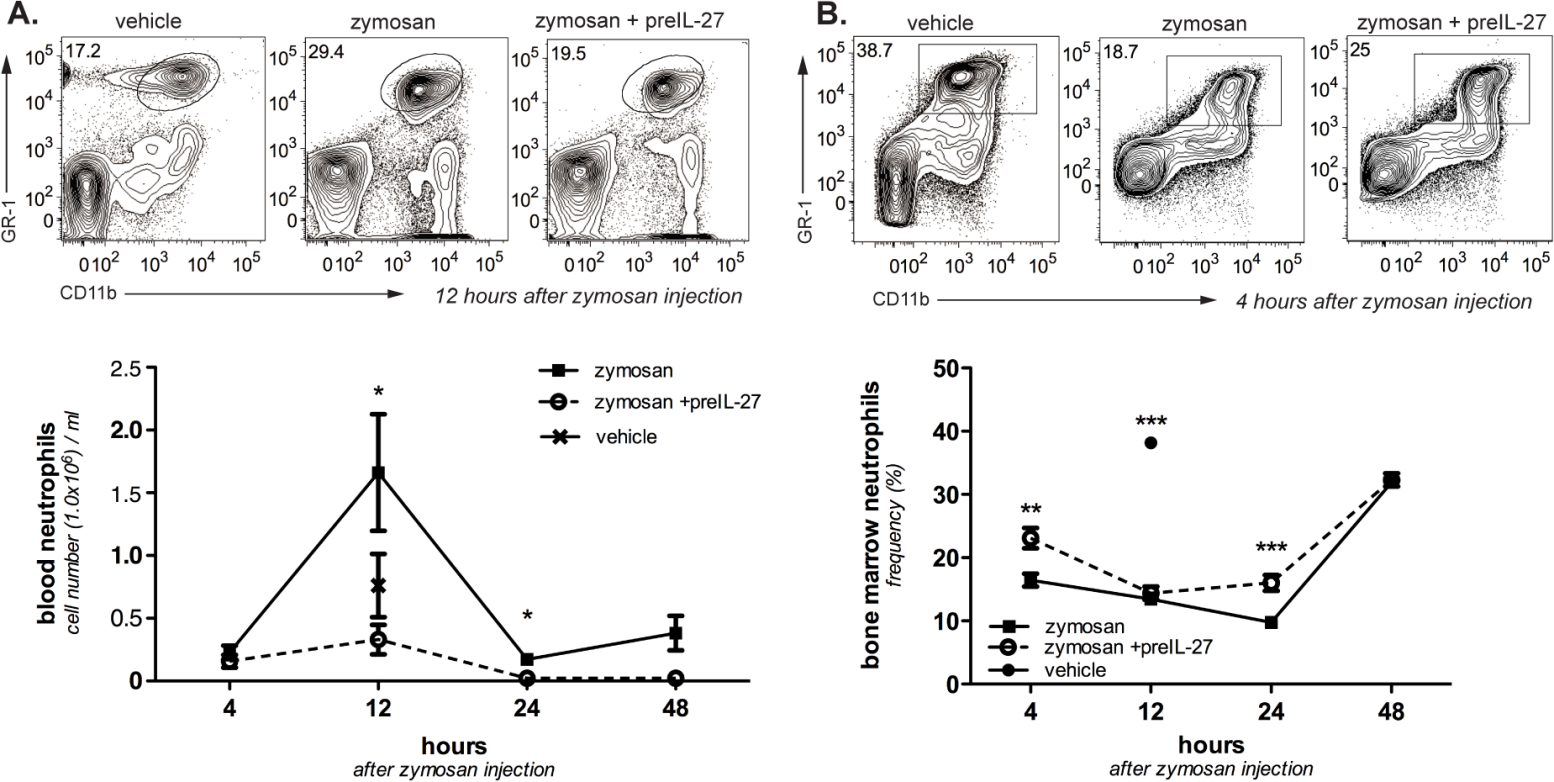
IL-27 pre-treatment decreases neutrophil accumulation in the murine blood and temporarily in the bone marrow during zymosan-induced peritonitis. Dot plots show the gating for neutrophils in the FACS analyses of blood cells and bone marrow cells (at at 12h and 4h after zymosan injection, respectively). Cell frequencies within the gate are given for the exemplary FACS plots. **A.** Absolute numbers of blood neutrophils in the blood of vehicle-treated animals (vehicle) 12h after injection and animals with or without pre-treatment with rIL-27 monitored 4–48h after injection of zymosan. Results from four different experiments are combined; given are means ± S.E.M (n = 8–11). **B.** Absolute numbers of neutrophils in the bone marrow of vehicle-treated animals (12h after vehicle injection) and 4–48h after injection of zymosan with or without pre-treatment with rIL-27. Results from four different experiments are combined; given are means ± S.E.M (n = 8–11). Mann–Whitney test was used to compare between groups, *p<0.05, ** p<0.01, ***p<0.005.

Cytometric analysis of peripheral blood leukocytes 12 h post- zymosan injection revealed a distinct increase of neutrophil numbers in the murine blood (Fig 3A), which parallels the observed 12 h peak in the peritoneal lavage (Fig 1B). Treatment with IL-27 12 h prior to zymosan injection resulted in a synchronous strong reduction of neutrophil cell numbers in the blood, even more pronounced compared to the drop in the peritoneal cavity. The strong reduction of blood neutrophils suggests that IL-27 acts not only on neutrophil recruitment but already at a systemic level.

Upon inflammation, neutrophils become mobilized from the bone marrow to enter the circulation at an enhanced rate [19]. Accordingly, we observed a marked decline in the frequency of bone marrow neutrophils already 4 h after zymosan application showing a fast mobilization of neutrophils after the inflammatory trigger (Fig 3B; zym 16.5% ± 1.0 versus 38.2% ± 0.9 in vehicle-injected animals). Interestingly, pre-treatment with IL-27 suppressed the mobilization from bone marrow resulting in significantly increased frequencies of neutrophils at 4 h and 24 h compared to the zymosan-only group and marginally different values at 12 h (4 h: zym 16.5% ± 1.0, preIL-27,23.1% ± 1.6; 12 h: zym 13.5% ± 0.8, preIL-27 14.4% ± 1.1; 24 h: zym 9.7% ± 0.8, preIL-27 16.0% ± 1.2). At 48 h after induction of peritonitis, neutrophil frequencies in the bone marrow of all zymosan-injected groups approached the levels of vehicle-injected animals (48 h: zym 32% ± 0.5, preIL-27 32% ± 1.0) indicating the cessation of the leukocyte mobilization from the bone marrow.

### IL-27 treatment influences chemokine but not cytokine levels in the peritoneal cavity

**Fig 4:**
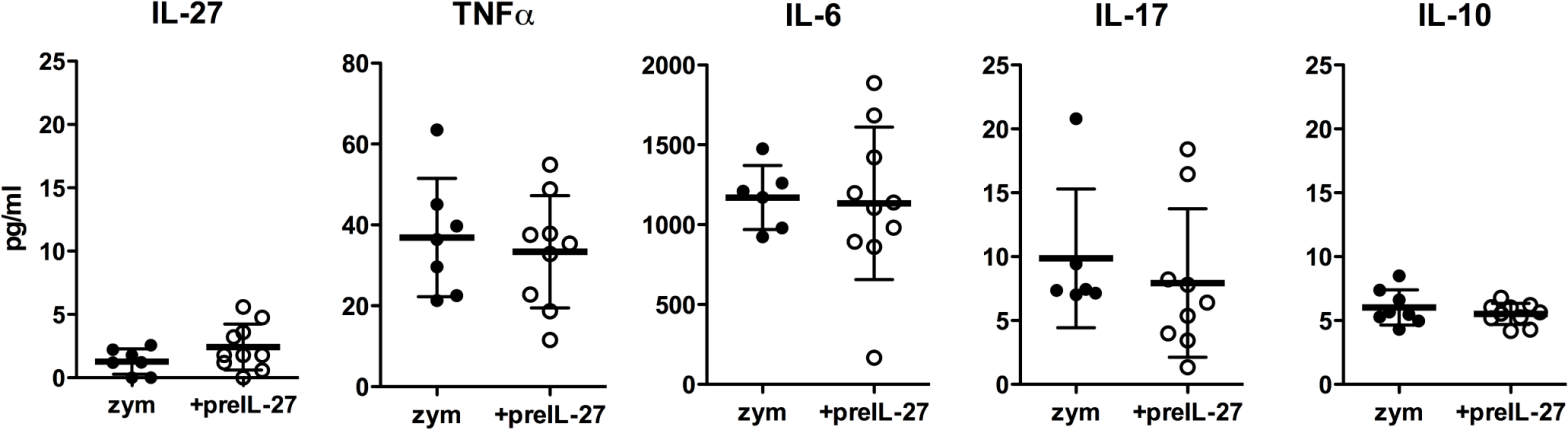
Comparable levels of cytokines in the peritoneal exudate. 12 h after induction of peritonitis the peritoneal cavity was flushed with 5 ml PBS and levels of the indicated cytokines were measured by multiplex bead array assay. Results from three different experiments are combined; given are means ± S.D (n = 6–10). Mann–Whitney test was used to compare between groups.

**Fig 5:**
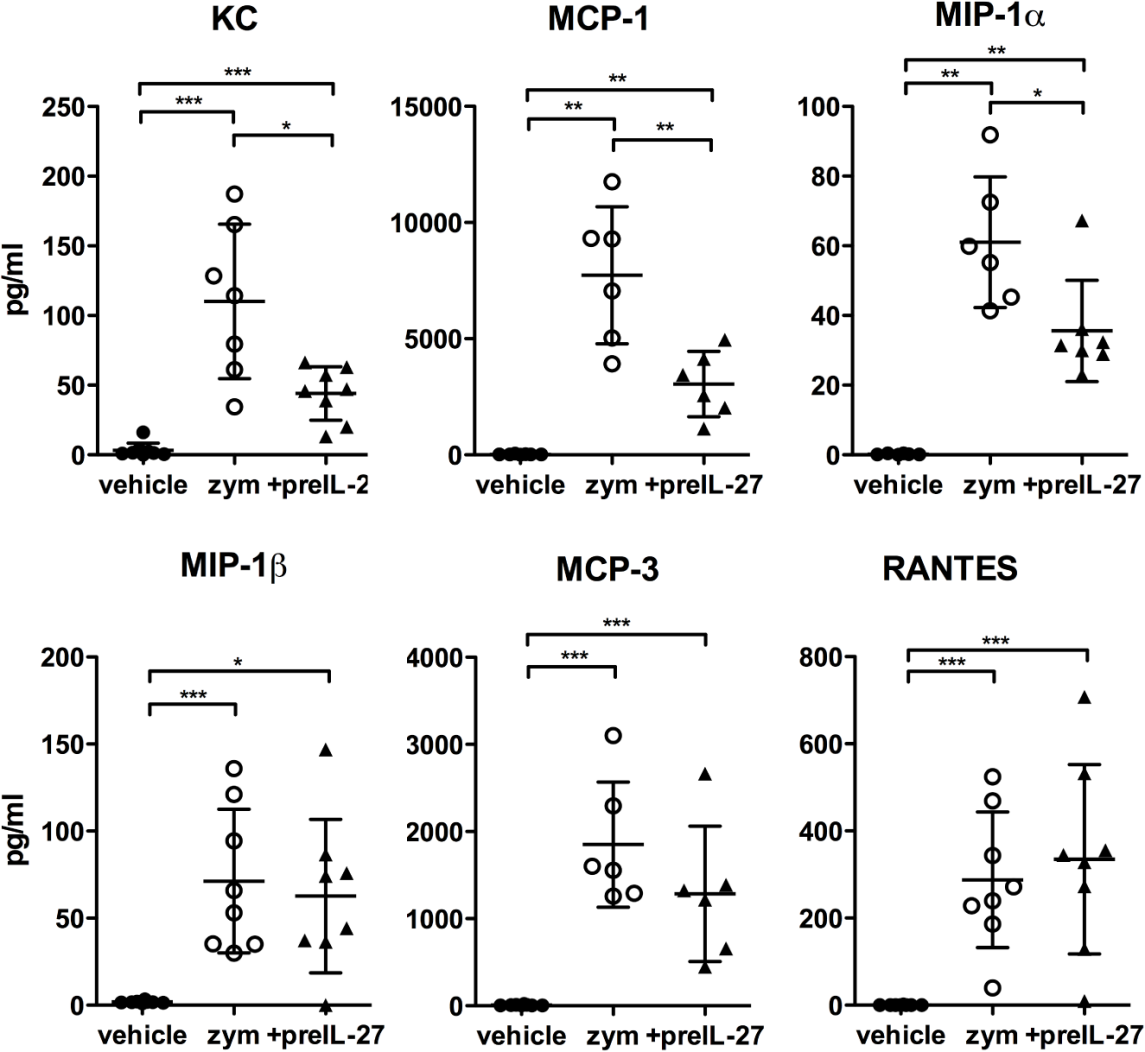
Decreased levels of KC, MCP-1 and MIP-1α in the peritoneal lavage after IL-27 pretreatment. 12 h after induction of peritonitis or vehicle injection, the peritoneal cavity was flushed with 5ml PBS and levels of the indicated chemokines were measured by multiplex bead array assay. Results are pooled from three independent experiments; given are means ± S.D (n = 6–8). Mann–Whitney test was used to compare between groups, *p<0.05, ** p<0.01, ***p<0.005.

To uncover whether the effects of IL-27 are direct or rely on the induction or suppression of other mediators, we analysed cytokine and chemokine levels in the peritoneal lavage by multiplex immunoassay 12 h after zymosan injection with or without pre-treatment with IL-27 (Figs 4 and 5). At this time point, IL-27 levels were already close to the detection limit, suggesting a rapid resorption of the injected cytokine (200 ng).

TNFα can be produced by macrophages, NK cells and T cells, but also by neutrophils [21]. Levels of TNFα were rather low and not significantly decreased upon IL-27 treatment. The pro-inflammatory cytokine IL-17 produced by T cells is strongly regulated by IL-27 [2, 3]. However, it can also be produced by NK cells and neutrophils. In the peritonitis model studied here, values of IL-17 were near the detection limit and the small decline upon IL-27 treatment was not significant. High levels were found for the acute-phase cytokine IL-6 that is mainly produced by macrophages; however, levels were not different between the groups.

IL-27 is a known inducer of the inhibitory cytokine IL-10 that in turn dampens inflammation and suppresses immune activation [6]. We found only minute levels of IL-10, which were not influenced by IL-27 pre-treatment, suggesting that IL-10 induction is not a major factor. Indeed, blocking with anti IL-10 had no effect on the suppressive action of IL-27 (data not shown).

In contrast to the unchanged cytokine levels, several chemokines were found to be reduced upon treatment with IL-27 (Fig 5). KC / CXCL1, a major neutrophil attractant corresponding to the human IL-8, showed significantly reduced levels in the IL-27 treated mice. Similarly, MCP-1 / CCL2 was strongly and MIP-1α / CCL3 markedly reduced. Weak or no effects were observed for MIP-1β /CCL4, MCP-3 / CCL7 and RANTES / CCL5. Vehicle-injected groups revealed values close to zero for each chemokine.

Significant chemokine levels in the blood plasma (12h) were only detectable upon zymosan treatment and mostly one order of magnitude lower (except KC and RANTES) than in the peritoneal lavage; however, IL-27 treatment resulted only in minor, non-significant decreases of KC or MCP-1 levels (S1 Fig). Thus, IL-27 treatment causes a rather discrete suppression of some circulating blood chemokines produced upon zymosan-induced inflammation, including the neutrophil-attracting KC. It might be mentioned that also the pattern of chemokines produced by in vitro cultured, zymosan-activated peritoneal macrophages is differentially and to a lesser extent influenced by IL-27 (S2 Fig), suggesting a more complex action of IL-27 on the cellular environment in vivo.

## Discussion

While the effects of IL-27 have been widely studied for the adaptive immune system, the regulatory role of IL-27 in the innate immune system is poorly understood. We investigated the role of IL-27 in sterile peritonitis induced by injection of zymosan into the peritoneal cavity. This well described model features all the mechanisms of an acute innate immune reaction, such as massive infiltration of neutrophils to the site of inflammation. As expected, we could observe a strong increase in neutrophil numbers in the peritoneal lavage after zymosan injection with a peak at 12 h. Interestingly, co-injection of IL-27 with zymosan did not influence the initial increase in neutrophil numbers, however, at later time points we observed a faster decline in neutrophils.

As IL-27 might not necessarily directly affect inflammatory cells but act via induction or repression of proteins yet to be synthesized, such as other cytokines, chemokines or inflammation-related adhesion molecules, more time could be required for IL-27 to become effective. Indeed, IL-27 treatment 12 h prior to zymosan injection led to a strong reduction in neutrophil numbers in the peritoneal cavity already at the peak of inflammation. Thus, IL-27 reduces the number of neutrophils in the peritoneal cavity at the peak of disease after a lag phase of several hours. Both applications of IL-27, either prior or simultaneously to the injection of zymosan, were accelerating the drop of neutrophils after the inflammatory peak. Whether the faster clearance of neutrophils from the peritoneal cavity indicates a role of IL-27 in the resolution of the inflammatory process [22], e.g. by stimulating their egress or their apoptosis, remains to be analysed.

Suppression of neutrophil accumulation in the peritoneal cavity could be due to inhibition of their recruitment from blood into peritoneal cavity, but also by regulation of their supply from the bone marrow. As is known, neutrophil numbers in the blood showed a strong increase after induction of peritonitis with a peak at 12h, similar to the increase in the peritoneum. Surprisingly, pre-treatment with IL-27 led also to a pronounced reduction in blood neutrophilia at the peak of inflammation. Thus, suppression of neutrophil accumulation in the peritoneal cavity is not simply due to inhibition of their extravasation from blood to peritoneum but rather seems to be controlled at the preceding level, the regulation of neutrophil numbers in the circulation. It might be mentioned that this early effect is unlikely to be due to an acceleration of the resolution process.

Neutrophils are mainly produced and stored in the bone marrow; upon inflammation, increased numbers are released into the circulation [19]. This was also found here: blood levels paralleled the kinetics of neutrophil accumulation in the peritoneum, while frequencies of neutrophils in the bone marrow were concomitantly reduced and later recovered. Pre-treatment with IL-27 led to a significantly decreased loss of neutrophils from the bone marrow as early as 4 h after induction of peritonitis. This suggests that the diminution both of neutrophil recruitment to the peritoneum and of blood levels by IL-27 treatment is caused by a decreased release of the cells from the bone marrow. We cannot exclude that IL-27, beyond regulating the exit from the bone marrow, also influences the migration of neutrophils into other organs that have been described as margination sites of neutrophils [19].

How IL-27 controls neutrophil release from the bone marrow is not clear so far. Inflammatory cytokines such as TNFα, known to augment release of neutrophils into the blood stream [19], were not affected by IL-27 treatment under our conditions. This is consistent with recent findings of Li et al. [19] showing that IL-27 treatment does not regulate TNF-levels in human neutrophils.

However, marked drops in the level of distinct chemokines in the peritoneal fluid were found here. Interestingly, a role of chemokines has also been discussed for the emigration process of leukocytes from the bone marrow [23]. We observed significantly reduced levels of KC, MCP-1 and MIP-1α in the peritoneal exudate at the inflammatory peak for IL-27 treated mice, whereas other chemokines showed no differences. Indeed, it has been shown previously that blockade of KC by monoclonal antibodies (mAbs) resulted in reduced neutrophil counts both in blood and peritoneal fluid. Vice versa, treatment with KC increased the number of neutrophils in blood and lowered their numbers in bone marrow [23]. These findings are reminiscent to findings of another study reporting chemerin-related agents to be effective in blocking neutrophil recruitment while also reducing, albeit less selectively, the levels of several cytokines and chemokines [24].

It is puzzling, however, that the reduction of KC, MCP-1 or MIP-1α in serum was less clear or not observed. Therefore, it remains unclear whether the strong decrease of these chemokines in the peritoneum is responsible for their decreased mobilization from the bone marrow or whether this only contributes to a reduced recruitment into the inflamed site. Alternatively, IL-27 might suppress other systemic mediators involved in neutrophil mobilization such as leukotrienes [19], or might act directly on constitutive chemokine- or adhesion molecule-regulated trapping of neutrophils in their bone marrow niches.

The delay in the biological effects of IL-27 suggests that a direct effect on zymosan-induced chemokine production is not the key mechanism. Rather, IL-27 could induce an intermediate suppressive factor, such as IL-10, which was, however, not changed. We could not find a change in the expression of the zymosan receptor, TLR2, or of CD11b as one important adhesion molecule of neutrophils under the influence of IL-27 (S3 and S4 Figs). Most likely, IL-27 could affect the final maturation and/or migratory activity of neutrophils in the bone marrow and thereby alter the levels of circulating neutrophils a couple of hours later.

Thus, the data of this study uncovers a novel mechanism how IL-27 is able to suppress inflammatory reactions by modulating the release of leukocytes from the bone marrow. Although we would not exclude additional effects of reduced chemokine levels on the extravasation of neutrophils towards the inflamed peritoneum, decreased neutrophil levels in the blood are sufficient to explain the reduced accumulation in the peritoneal fluid.

In conclusion, this study reveals that IL-27 has a distinct impact on the innate arm of inflammatory responses, which are independent from its well described effects on T cells. The results extend our previous findings in an influenza infection model [10] and suggest that application of IL-27 might represent an interesting therapeutic option to control leukocyte-mediated immunopathology in infectious or sterile inflammatory diseases. In addition, our findings uncover a novel mechanism of anti-inflammatory cytokines by showing that IL-27 is able to suppress inflammatory reactions by stopping the enhanced release of neutrophils from the bone marrow.

## Supporting Information

S1 Fig Reduced levels of KC and MCP-1 in the blood plasma after IL-27 pretreatment. Multiplex bead array assay was used to detect levels of indicated chemokines in the blood plasma obtained 12h after induction of peritonitis. Non-treated animals (NT) received neither zymosan nor IL-27. Values are means ± S.E.M. Results are pooled from two to three independent experiments (n = 3–6). Mann–Whitney test was used to compare between groups.
(TIF)

S2 Fig Levels of chemokines after in vitro stimulation with zymosan are not changed upon IL-27 pretreatment. Peritoneal exudate cells were isolated and cultured at a final concentration of 2 x 10^6^ cells/ml cRPMI (RPMI 1640 (Gibco®) plus 10% FCS (vol/vol) and antibiotics) at 37°C and 5% CO_2_. PreIL-27 samples were treated with IL-27 (50 ng/ml) 12h before zymosan stimulation (50 μg/ml). Zymosan group received no treatment and PBS group no zymosan stimulation. Supernatant was harvested 12h after zymosan treatment and a multiplex bead array assay was performed to detect chemokine levels. The graph shows results from one experiment (n = 4, for each group). Mann-Whitney test was used to compare between groups.
(TIF)

S3 Fig TLR-2 expression on peritoneal macrophages after in vitro stimulation with zymosan is not changed upon IL-27 treatment. Peritoneal exudate cells were isolated and cultured at a final concentration of 2 x 10^6^ cells/ml cRPMI (RPMI 1640 (Gibco®) plus 10% FCS (vol/vol) and antibiotics) at 37°C and 5% CO2. PreIL-27 samples were treated with IL-27 (200 ng/ml) 12h before zymosan stimulation (100 μg/ml). Zymosan group received no treatment and PBS group no zymosan stimulation. 12h after zymosan treatment cells were harvested, washed and stained for FACS analysis. The graph shows the mean flourecence intensity (MFI) of the TLR-2 signal of peritoneal macrophages (gated on CD11b+F4/80+ cells). Depicted are results from one experiment (n = 4, for each group).
(TIF)

S4 Fig Similar levels of CD11b on blood neutrophils after IL-27 pretreatement. 12h after induction of peritonitis, mice were sacrificed and blood samples were stained for FACS analysis. The graph shows the MFI of the CD11b signal of blood neutrophils (gated on GR-1^high^ cells). Results are pooled from three independent experiments (n = 9–10). Mann–Whitney test was used to compare between groups.
(TIF)

S1 Table Underlying data behind figures. (DOCX)
