# Supplementary figures and images for: Anti-Inflammatory Effects of IL-27 in Zymosan-Induced Peritonitis: Inhibition of Neutrophil Recruitment Partially Explained by Impaired Mobilization from Bone Marrow and Reduced Chemokine Levels
Source: PLoS One. 2015 Sep 11;10(9):e0137651. doi: 10.1371/journal.pone.0137651 (PMC4567321; doi:10.1371/journal.pone.0137651)

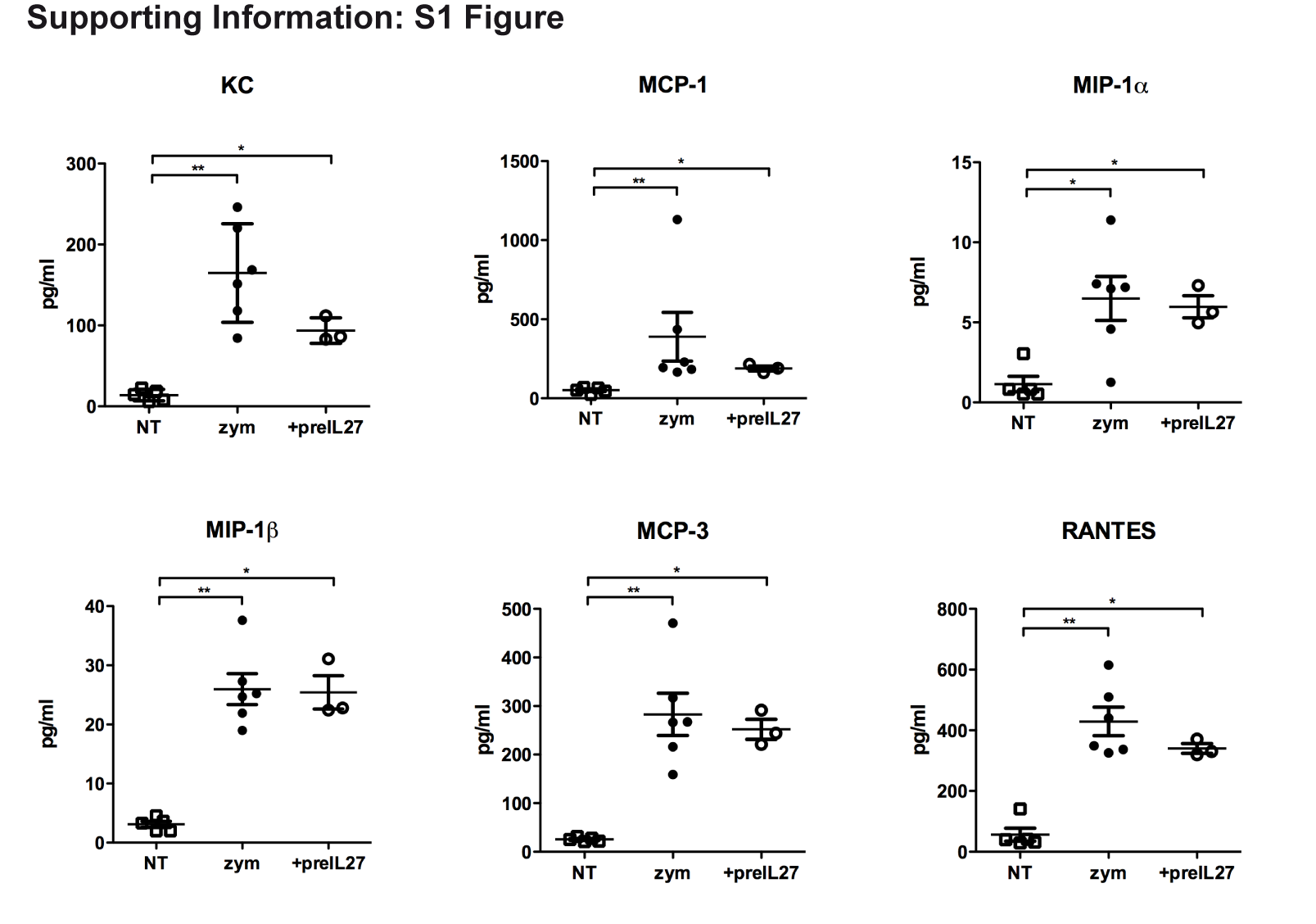

Supplement: S1 Fig — Multiplex bead array assay was used to detect levels of indicated chemokines in the blood plasma obtained 12h after induction of peritonitis. Non-treated animals (NT) received neither zymosan nor IL-27. Values are means ± S.E.M. Results are pooled from two to three independent experiments (n = 3–6). Mann–Whitney test was used to compare between groups. (TIF) [file pone.0137651.s001.tif]

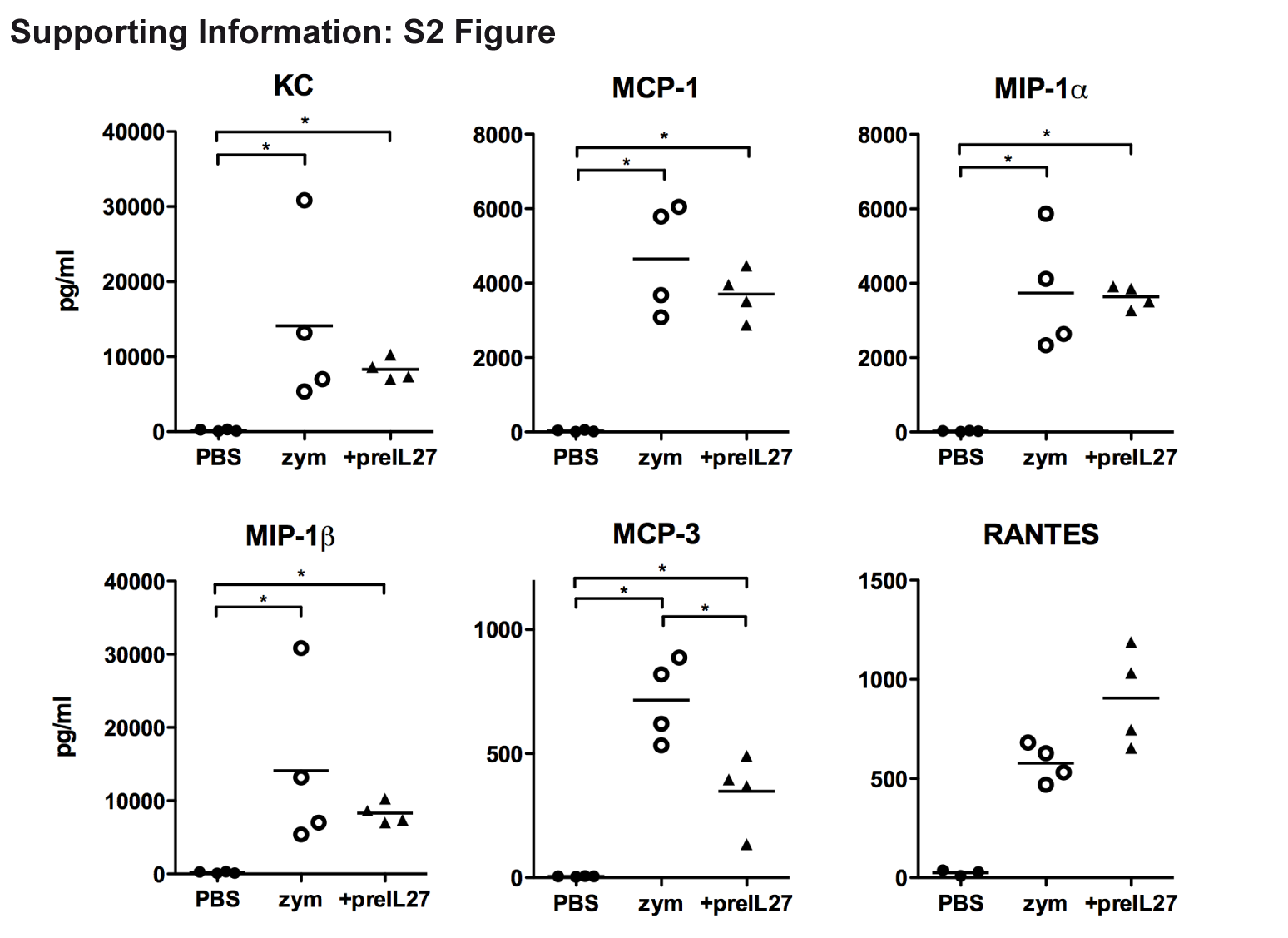

Supplement: S2 Fig — Peritoneal exudate cells were isolated and cultured at a final concentration of 2 x 10^6^ cells/ml cRPMI (RPMI 1640 (Gibco®) plus 10% FCS (vol/vol) and antibiotics) at 37°C and 5% CO2. PreIL-27 samples were treated with IL-27 (50 ng/ml) 12h before zymosan stimulation (50 μg/ml). Zymosan group received no treatment and PBS group no zymosan stimulation. Supernatant was harvested 12h after zymosan treatment and a multiplex bead array assay was performed to detect chemokine levels. The graph shows results from one experiment (n = 4, for each group). Mann-Whitney test was used to compare between groups. (TIF) [file pone.0137651.s002.tif]

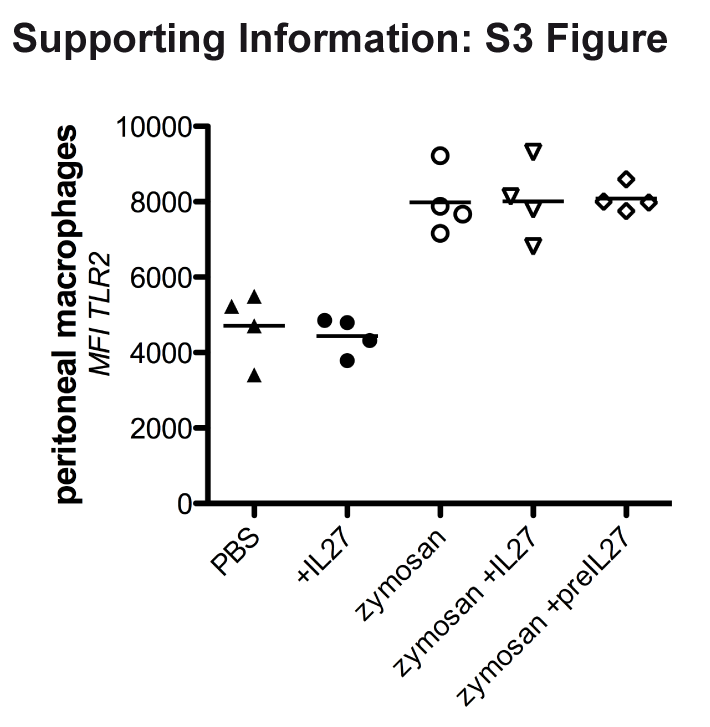

Supplement: S3 Fig — Peritoneal exudate cells were isolated and cultured at a final concentration of 2 x 10^6^ cells/ml cRPMI (RPMI 1640 (Gibco®) plus 10% FCS (vol/vol) and antibiotics) at 37°C and 5% CO2. PreIL-27 samples were treated with IL-27 (200 ng/ml) 12h before zymosan stimulation (100 μg/ml). Zymosan group received no treatment and PBS group no zymosan stimulation. 12h after zymosan treatment cells were harvested, washed and stained for FACS analysis. The graph shows the mean flourecence intensity (MFI) of the TLR-2 signal of peritoneal macrophages (gated on CD11b+F4/80+ cells). Depicted are results from one experiment (n = 4, for each group). (TIF) [file pone.0137651.s003.tif]

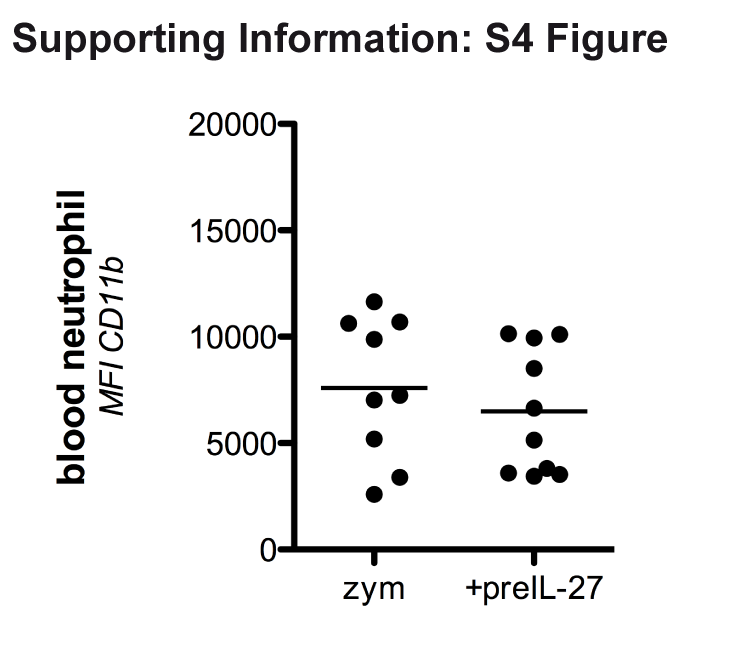

Supplement: S4 Fig — 12h after induction of peritonitis, mice were sacrificed and blood samples were stained for FACS analysis. The graph shows the MFI of the CD11b signal of blood neutrophils (gated on GR-1high cells). Results are pooled from three independent experiments (n = 9–10). Mann–Whitney test was used to compare between groups. (TIF) [file pone.0137651.s004.tif]
