# Supplementary material for: Anti-Inflammatory Effects of IL-27 in Zymosan-Induced Peritonitis: Inhibition of Neutrophil Recruitment Partially Explained by Impaired Mobilization from Bone Marrow and Reduced Chemokine Levels
Source: PLoS One. 2015 Sep 11;10(9):e0137651. doi: 10.1371/journal.pone.0137651 (PMC4567321; doi:10.1371/journal.pone.0137651)
Supplement: S1 Table — (DOCX) [file pone.0137651.s005.docx]

**Supporting Information: S5 Tables**

**Fig. 1B – Single values**

|  | **zymosan** | | | | | | | | | |
| --- | --- | --- | --- | --- | --- | --- | --- | --- | --- | --- |
| 4h | 3920000 | 5580000 | 6150000 | 6360000 | 1400000 | 53600 | 1590000 |  |  |  |
| 12h | 9730000 | 11000000 | 11100000 | 7190000 | 8050000 | 8750000 | 9400000 | 7720000 |  |  |
| 24h | 9720000 | 8680000 | 8370000 | 3440000 | 6590000 | 4990000 | 5960000 | 4470000 | 1900000 |  |
| 48h | 3040000 | 2500000 | 3270000 | 5690000 | 2760000 | 2190000 |  |  |  |  |
|  |  |  |  |  |  |  |  |  |  |  |
|  | **zymosan +IL-27** | | | | | | | | | |
| 4h | 10400000 | 533000 | 7110000 | 7640000 | 776000 | 3550000 | 3350000 |  |  |  |
| 12h | 10900000 | 13300000 | 10300000 | 9040000 | 4490000 | 8890000 | 5310000 | 9430000 |  |  |
| 24h | 5110000 | 2580000 | 5570000 | 2840000 | 558000 | 3130000 | 3600000 | 4660000 | 9890000 | 90700 |
| 48h | 2460000 | 1800000 | 1750000 | 1530000 | 1170000 | 1140000 | 2110000 |  |  |  |
|  |  |  |  |  |  |  |  |  |  |  |
|  | **zymosan + pre IL-27** | | | | | | | | | |
| 4h | 1710000 | 3510000 | 231000 | 3340000 |  |  |  |  |  |  |
| 12h | 4110000 | 4480000 | 8170000 | 4610000 | 2930900 | 2230200 | 2580600 | 2656500 |  |  |
| 24h | 2360000 | 5110000 | 4820000 | 3630000 |  |  |  |  |  |  |
| 48h | 3130000 | 2680000 | 71600 | 865000 |  |  |  |  |  |  |
|  |  |  |  |  |  |  |  |  |  |  |
|  | **vehicle** | | | | | | | | | |
| 12h | 172.9688 | 2111.595 | 107250 | 73255.81 | 11862.5 | 11297.92 | 6566.667 | 5100 |  |  |

**Fig. 1C – Single values**

|  | **zymosan** | | | | | | | | | | | |
| --- | --- | --- | --- | --- | --- | --- | --- | --- | --- | --- | --- | --- |
| 4h | 237000 | 87300 | 347000 | 701000 | 532000 | 1150000 | 498000 |  |  |  |  |  |
| 12h | 1150000 | 1270000 | 1050000 | 1380000 | 1350000 | 1740000 | 1040000 | 1270000 |  |  |  |  |
| 24h | 2100000 | 1560000 | 1730000 | 1720000 | 2580000 | 3300000 | 3440000 | 297190 | 793000 | 439110 | 345840 | 155430 |
| 48h | 2030000 | 2210000 | 1890000 | 2470000 | 1920000 | 2030000 |  |  |  |  |  |  |
|  |  |  |  |  |  |  |  |  |  |  |  |  |
|  | **zymosan +IL-27** | | | | | | | | | | | |
| 4h | 160000 | 853000 | 714000 | 912000 | 50300 | 1020000 | 849000 |  |  |  |  |  |
| 12h | 1350000 | 858000 | 1380000 | 2090000 | 1720000 | 2110000 | 1360000 | 825000 |  |  |  |  |
| 24h | 3210000 | 682000 | 666000 | 3160000 | 1420000 | 4400000 | 1950000 |  |  |  |  |  |
| 48h | 1520000 | 1970000 | 2020000 | 2980000 | 1590000 | 2170000 | 2060000 |  |  |  |  |  |
|  |  |  |  |  |  |  |  |  |  |  |  |  |
|  | **zymosan + pre IL-27** | | | | | | | | | | | |
| 4h | 413000 | 356000 | 152000 | 396000 |  |  |  |  |  |  |  |  |
| 12h | 795000 | 920000 | 1400000 | 661000 |  |  |  |  |  |  |  |  |
| 24h | 2380000 | 2540000 | 4890000 | 2170000 | 618270 | 421260 | 497280 | 391860 |  |  |  |  |
| 48h | 3930000 | 1600000 | 2100000 | 754000 |  |  |  |  |  |  |  |  |
|  |  |  |  |  |  |  |  |  |  |  |  |  |
|  | **vehicle** | | | | | | | | | | | |
| 12h | 85218.75 | 487598.7 | 1293750 | 601744.2 | 255666.7 | 144375 | 247666.7 | 232875 |  |  |  |  |

**Fig. 3A – Single values**

|  | **zymosan** | | | | | | | | | | |
| --- | --- | --- | --- | --- | --- | --- | --- | --- | --- | --- | --- |
| 4h | 176000 | 359000 | 328000 | 423000 | 153000 | 27000 | 130000 |  |  |  |  |
| 12h | 2780000 | 1570000 | 2060000 | 5310000 | 2790000 | 254000 | 103000 | 1500000 | 405056.3 | 841890 | 661603.8 |
| 24h | 204000 | 203000 | 104000 | 64000 | 289000 | 276000 | 369000 | 366000 | 3310 | 15000 | 4680 |
| 48h | 852000 | 670000 | 686000 | 412000 | 19300 | 25100 | 13900 |  |  |  |  |
|  |  |  |  |  |  |  |  |  |  |  |  |
|  | **zymosan + pre IL-27** | | | | | | | | | | |
| 4h | 97400 | 239000 | 46800 | 246000 |  |  |  |  |  |  |  |
| 12h | 65100 | 154000 | 101000 | 162000 | 811395 | 735110 | 286235 |  |  |  |  |
| 24h | 10100 | 67000 | 1820 | 21100 | 50678.57 | 18571.43 | 28571.43 | 406.9767 | 390.2344 |  |  |
| 48h | 22000 | 5120 | 32800 | 29800 |  |  |  |  |  |  |  |
|  |  |  |  |  |  |  |  |  |  |  |  |
|  | **vehicle** | | | | | | | | | | |
| 12h | 308550 | 590400 | 410400 | 469800 | 1968600 | 814000 |  |  |  |  |  |

**Fig. 3B – Single values**

|  | **zymosan** | | | | | | | | | | | |
| --- | --- | --- | --- | --- | --- | --- | --- | --- | --- | --- | --- | --- |
| 4h | 12.4 | 13 | 16.3 | 19.2 | 18.1 | 17.7 | 18.5 |  |  |  |  |  |
| 12h | 14.2 |  | 14.8 | 13.3 | 12.4 | 17.2 | 9.49 | 12.4 | 13.9 |  |  |  |
| 24h | 8.54 | 13.1 | 10.3 | 11.6 | 7.44 |  | 8.17 | 14.3 | 10.4 | 9.03 | 5.6 | 8.65 |
| 48h | 31.9 | 31.1 | 32.7 |  |  |  |  |  |  |  |  |  |
|  |  |  |  |  |  |  |  |  |  |  |  |  |
|  | **zymosan + pre IL-27** | | | | | | | | | | | |
| 4h | 25 | 19.2 | 18.5 | 22.2 | 19.7 | 32.5 | 24.9 | 22.7 |  |  |  |  |
| 12h | 12.1 | 20.3 | 12.7 | 12.2 | 11.8 | 13.6 | 15.9 | 16.5 |  |  |  |  |
| 24h | 12.4 | 12.5 | 14.9 | 14.7 | 19.3 |  | 13.7 | 24 | 17.3 | 15.3 |  |  |
| 48h | 30.8 | 31.8 | 31.3 | 35.3 |  |  |  |  |  |  |  |  |
|  |  |  |  |  |  |  |  |  |  |  |  |  |
|  | **vehicle** | | | | | | | | | | | |
| 12h | 37.6 | 36.6 | 39.4 | 39.2 | 43.2 | 36.1 | 34.7 | 38.4 |  |  |  |  |
